# Use of Tactile Contact Accompanying Health Promotion Messages During Routine Health & Physical Examinations: A Technique for Improving Compliance

**Published:** 2022-02-09

**Authors:** Ralph Jay Johnson

**Affiliations:** 1 Research PA-C/Research Coordinator University of Texas MD Anderson Cancer Center

**Keywords:** Tactile contact, touching, program compliance, anchoring, treatment compliance, health improvement, health promotion

## Abstract

A daunting challenge for health providers and medical practitioners is communicating the vital importance of health promotion and medical treatment adherence and compliance. This article is an evidence-based, best-practices commentary advocating the use of touch-accompanied verbal suggestions during the touching portions of routine, near-universal Health & Physical examinations. Notional case examples are presented; based on the professional literature, underlying Behavioral Mechanics are discussed. Touch-accompanied verbal health promotion messages skillfully deployed in routine Health & Physical examinations offer a non-harmful and efficient technique to synergistically and substantially enhance the probability of patient compliance with health improvement and medical treatment regimens. Though it is not a magic panacea, the public health applications, extensions and benefits are incalculable in terms of healthy behavior adoption. Additionally, if deftly conducted in accordance with best practices, it has the potential to greatly improve practitioner-patient relations and increase patient satisfaction. Further avenues of research inquiry are considered.

## Introduction

A Health and Physical (i.e., “H&P”) examination is commonly considered a universal medical procedure performed as part of most face-to-face heath care encounters in which medical practitioners interview and examine patients for any medical signs and symptoms of medical conditions.[1–4] Even as telemedicine encounters become common and accepted, almost everyone invariably at some point must meet face-to-face with a medical practitioner for an H&P.[5–7] This examination typically includes a series of questions regarding patients’ medical histories, followed by tactically-based examinations on reported symptoms. [1,8,9] The aim naturally is to preliminarily rule-out a diagnosis, order more definitive objective/empirical tests, and formulate a treatment plan.[9,10] Substantial research supports the contention that patients universally expect these examinations; these examinations are variously depicted as a necessary ritual that plays a vital and substantial role in fostering the medical practitioner- patient relationship, which improves subsequent medical encounters.[11–13] When these examinations are not performed, patients may feel the validity of their illness and treatment are insufficient and slighted, which then mars the medical practitioner-patient relationship.[11,13,14, 15]

The H&P may involve standard tests (e.g., vital signs such as temperature, heart rate, respiration, blood pressure etc.).[16] It also involves the medical practitioner using various senses, especially hearing and particularly touch, starting at the head of the patient and ending at the toes.[9,16,17] Medical practitioners examine patients visually and tactically through inspection, palpation, percussion, auscultation, and even manipulation such as shaking.[17,18] During touching, there is ample opportunity for verbal communication between medical practitioners and patients.[9,16,19,20] Notably, whereas in some contexts touching is prohibited and taboo, in the context of an authoritative H&P, medical practitioners’ legitimate and acceptable touching is expected and encouraged.[11–15][17] Indeed, during this process, beneficial health promotion messages can be delivered subtly to patients to powerfully improve their treatment compliance and outcomes.[21] This is because the messages are delivered in a particular manner and simultaneously combined with touch in a legitimate, authoritative medical encounter.[21–23]

The purpose of this commentary / review is to advance the notion of deploying subtle beneficial health improvement messages, accompanied by appropriate touching, during the portion of H&Ps that involve medical practitioner’s touching assessments. This is done in order to augment more formal and even written health instructions, and thus to achieve beneficial outcomes for patients. These may include a strengthened therapeutic medical practitioner- patient alliance.

## Background

A traditionally challenging task for medical practitioners is communicating the importance of treatment plan compliance, including but not limited to healthcare follow-up—be it therapeutic regimen adherence, medication schedules, diet restrictions, or follow-up visits[24–30]. Additionally, one of the most primal and powerful modes of communication is touch; research conclusively suggests that along with a suggestion, request, or directive, touch has a synergistic effect on reciprocal compliance.[31, 32] In a series of foundational field experiments in varied natural settings over several years, Gueguen et al. [33–37] clearly demonstrated that brief touching with a direct gaze, when accompanied by a request, had a maximally positive influence on compliance—whether or not the subjects even were aware they had been touched. Hornik;[38] Smith; Gier; and Willis;[39] Willis and Hamm; [40] and Crusco and Wetzel found that tactile contact enhanced spontaneous compliance or improved compliance— even when no explicit verbal request was made. Johnson(2021)[42] conducted a field experiment with street drug users attending a health improvement outreach program and found a statistically significant difference; those who were socially appropriately touched and requested with direct gaze to continue to attend the program were more likely to do so than those who were not touched but similarly requested. This body of conclusive research convincingly demonstrates the viability of a similar best-practices practical extension and application during the touching portion of the H&P encounter. That is, socially acceptable touch in an H&P encounter can synergistically enhance the potential for compliance even with recalcitrant patients if accompanied by a subtle yet direct suggestion or request. Additionally, the minimally non-intrusive but efficient technique is relatively innocuous and not harmful or intrusive; it demonstrates genuine interest and appreciation of patients, thus improving the medical practitioner-patient relationship.[26,32,41,43,44, 45]

### Step-by-Step Technique

The following are two descriptive step-by-step examples.

Example 1

1. During a routine examination, a medical practitioner smelled tobacco smoke and residue on a patient and inquired about their smoking history. The patient admitted to smoking but expressed a desire to quit, and stated having tried several times yet not having the willpower to do so;
2. while auscultating with a stethoscope and listening to the patient’s respirations and touching the patient, that is, holding the stethoscope to the patient’s body;
3. the medical practitioner looked into the patient’s eyes; and,
4. very gently and subtly says, “You will get the willpower to quit smoking.”

Or,

Example 2

1. A physician has a patient where it is imperative that they return for follow-up appointments, but the patient expressed ambivalence and vacillated about doing so;
2. during a routine H&P, the practitioner is palpating their extremities for abnormalities while the patient is reclining on the examination table;
3. the physician looks the patient directly in the eyes; and,
4. subtly yet mildly directs, “You will come back for your follow-up appointment.”

In each case, there are pre-existing histories from which the practitioners draw, and the verbal suggestions are integrated with appropriate assessment touching during the H&P. The medical practitioners’ verbal suggestions constitute a clear, concise, understandable reframing of patients’ words only into a gentle yet firm directive or command with direct gaze. Research confirms that verbal communications accompanying touch should work optimally when delivered in a gentle, nonjudgmental, subtle yet directing / instructing manner.[46] They are centered on reframing patients’ own expressions of thoughts, feelings, preferences, observations, and expectations; the practitioner serves as an interpreter and synthesizer as seen in the above examples.[46, 47] This can be promoted by an initial exchange of information between practitioner and patient, usually during the Medical History portion of the H&P. [47–51] For example:

Patient (during Medical History interview) spontaneously expresses: “I am really having a lot of trouble keeping the pounds off.”
Medical Practitioner (during Auscultation Touching) reframes/synthesizes: “You will lose weight.” (Direct gaze, if possible, is ideal.)

That is in accordance with best practices. To be maximally effective, suggestions should be brief, clear, concise, structured, and prescriptive. Instructive or directive messages are reframed from patients’ own self-centered expositions and expressions of concerns to an attentive practitioner [46–51][52]. Also, the practitioners’ reframing shows patients the practitioner is attentive.[48–53] It is simple yet very effective, when accompanied by touch.[33–42]

### Behavioral Mechanics Explained

Though the behavioral mechanics of touch and suggestibility are not well-understood, the nursing profession has long been aware of and asserted the power of the therapeutic alliance between touch, accompanied by instructive verbal instruction, in transforming behavior.[21,55] The nursing literature has asserted that the more a patient needs instructive help, the more they help by touching accompanied by supportive communication, the better the results.[56] (Note: Supplementary direct gaze merely quickly alerts recipients’ attentiveness to touch and suggestion without (re-)focusing attention.[21]) According to the nursing literature, touch and verbal communication are powerful, complementary and synergistic in their intended outcome—literally tethering the mind of the nurse to the patient’s mind and body.[56, 57, 22]* The nursing literature claims that appropriate gentle touch has the formidable potential to soothe and heal (i.e., “laying on of hands”), allowing patients pause and permission to apprehend and accept accompanied communication and instruction.[58]

Montagu (1958)[58] notes that the oldest and most sensitive sense organ—the skin—is the first and paramount medium for communication. Touch is a more powerful (re-)enforcer than the content of language; when combined, the two become so commanding they cannot be denied. Appropriate touch in the H&P is defined as diagnostic assessment through inspecting, palpating, auscultating, probing, exploring, and manipulating the body object. If combined with verbal health improvement messages, it has the potential to synergize the healing communication process.[58] Skillfully deployed by the examiner and welcomed by the patient as a full participant in the communication, it triggers a concordance, acceptance, and internalization of accompanied instructions for response and compliance with the health promotion messages.[58,59,60]** Therefore, when a skilled medical practitioner incorporates legitimate, acceptable touch accompanied by health promotion messages in the setting of the H&P encounter, it has great potential not only for reinforcing positive healthy practices but also the practitioner-patient relationship.[61]

Research has shown that collection and reinforcement of patient-centered expositions is related to a more formative medical practitioner-patient relationship and higher patient satisfaction with their treatment—if only in that it shows the medical practitioner is listening to the patient.[48–53][62] That is, research shows there is a mutual concordance between effective communication, compliance, and patient satisfaction. Combined with touch, it can become that much more significant, consuming, and beneficial.

## Conclusion

This report advanced the idea of using the “touching portion” of the medical H&P as an ideal opportunity for health/medical practitioners to combine and deliver subtle beneficial health and treatment improvement messages via touching (and preferably with direct gaze) in achieving maximum effectiveness and augmenting more formal and written instructions—even helping to cultivate the therapeutic medical practitioner-patient alliance. Put differently, if skillfully deployed in routine Health and Physical examinations, touch-accompanied verbal health promotion messages offer an inexpensive, minimally invasive, and non-harmful technique to substantially augment health improvement, medical treatment regimens, enhance patient satisfaction, and advance public health initiatives through healthy behaviors adoption. Its beneficial uses in the health field are practically limitless. Though exactly how it works may still be a mystery, what matters is that it does work, often powerfully—and that it can be marshalled into H&Ps for patients’ well-being. What is also unknown is the extent to which this technique already is being used, perhaps by the more successful practitioners. It is plausible that touch and verbal communication are so common a part of the H&P process that it has been overlooked in terms of designing meaningful evaluative studies.

Given that communication in such encounters is always two-way, a question might be to what extent and how medical practitioners are themselves affected by it.[63] Also, with issues about the appropriateness of touch in medical encounters, particular contexts under which this technique can be optimally rendered or should be avoided should be considered (e.g., cultural / gender prohibitions). Specifically, prescriptions and prohibitions must be codified regarding when it should be used and when it should not be used. [61]

The deft and skilled use of touch should be considered in health care professionals’ academic curricula and residencies, and this should be guided by best practices informed by scientific research.[19,64] To reiterate, any ethical qualms first must be considered; just because touch can be optimally used in an H&P setting does not necessarily mean it should be used. However, while sophisticated technologies can be relied upon for diagnosis and treatment, interpersonal communication is the primary tool to influence patients’ health behaviors. As such, every synergist such as touch-accompanied communication should be considered in the interest of patients’ well-being.[50] And despite a general awareness that touch accompanied by verbal directive communication has potentially powerful implications for positive behavioral outcomes, particularly in H&Ps, it is clearly under-taught across the medical professions where it is most likely to be used. .[19,64] As with any skillset, understanding, training, and practice can only improve it.

## Data Availability

Yes, publications and sources are available on-line or provided by author upon request.

## Footnotes

* (Hypnotherapists have long recognized the “anchoring” power of touch accompanied by a subtle yet commanding suggestion. Touch refocuses attention to the area affected, thereby alerting the state of consciousness and making recipients more suggestable and even setting into motion internal auto-suggestion. Hypnotherapists’ common parlance for this auto- suggestive process is “dropping an anchor.” [65,66] This is done without the lengthy process of hypnotherapeutic induction. Note: The commentary herein is not advocating the hypnotizing of patients, nor is the process described herein hypnosis.)

** (Note: Auto-suggestive properties are commonly referred to in lay terms as “putting a bug in someone’s ear.”[67,68] In the Transtheoretical Model Stages of Change, this also may be referred to as enabling a shift from Pre-contemplation to Contemplation, and eventually, Action. [69])
